# Litter loss triggers estrus in a nonsocial seasonal breeder

**DOI:** 10.1002/ece3.935

**Published:** 2014-01-03

**Authors:** Sam MJG Steyaert, Jon E Swenson, Andreas Zedrosser

**Affiliations:** 1 Institute of Wildlife Biology and Game Management, University of Natural Resources and Life SciencesVienna, A-1180, Austria; 2 Department of Ecology and Natural Resource Management, Norwegian University of Life SciencesÅs, NO-1432, Norway; 3 Norwegian Institute for Nature ResearchTrondheim, NO-7485, Norway; 4 Department of Environmental and Health Studies, Telemark University CollegeBø, NO-3800, Norway

**Keywords:** Lactational anestrus, reproductive fate, reproductive strategy, sexual selection, sexually selected infanticide, *Ursus arctos*

## Abstract

Sexually selected infanticide (SSI) is often presumed to be rare among seasonal breeders, because it would require a near immediate return to estrus after the loss of an entire litter during the mating season. We evaluated changes in reproductive strategies and the reproductive fate of females that experienced litter loss during the mating season in a seasonal breeder with strong evidence for SSI, the brown bear. First, we used a long-term demographic dataset (1986–2011) to document that a large majority of females (>91%) that lose their entire litter during the mating season in fact do enter estrus, mate, and give birth during the subsequent birthing season. Second, we used high-resolution movement data (2005–2011) to evaluate how females changed reproductive strategies after losing their entire litter during the mating season. We hypothesized that females would shift from the sedentary lifestyle typical for females with cubs-of-the-year to a roam-to-mate behavior typical for receptive females in no more than a few (∼3) days after litter loss. We found that females with cubs-of-the-year moved at about 1/3 of the rate and in a less bimodal diurnal pattern than receptive females during the mating season. The probability of litter loss was positively related with movement rate, suggesting that being elusive and sedentary is a strategy to enhance cub survival rather than a relic of cub mobility itself. The movement patterns of receptive females and females after litter loss were indistinguishable within 1–2 days after the litter loss, and we illustrate that SSI can significantly reduce the female interbirth interval (50–85%). Our results suggest that SSI can also be advantageous for males in seasonally breeding mammals. We propose that infanticide as a male reproductive strategy is more prevalent among mammals with reproductive seasonality than observed or reported.

## Introduction

Reproductive strategies have evolved through natural and sexual selection as adaptations to optimize lifetime reproductive success in a certain environmental setting (Pianka 1976). These adaptations can be physiological (e.g., estrus cycling, the mechanism of implantation or ovulation), morphological (e.g., sexual ornaments, body size), and behavioral (e.g., contest competition for mates, multimale mating) and can vary within and between the sexes (Gross 1996). Ultimately, a species' suite of reproductive strategies determines its mating system (Shuster and Wade 2003).

Lactation is a universal mammalian attribute and restricts parental care predominantly to females in mammals (Millar 1977; Shuster and Wade 2003). Because lactation is energetically very costly and is of crucial importance for offspring fitness, strong selective pressures act upon characteristics of lactation and associated reproductive traits (Millar 1977; Schulz and Bowen 2005). In many species, mammary stimulation inhibits estrus through the production of hormones, such as prolactin and oxytocins (i.e., lactational anestrus) (Kann and Martinet 1975; Asa 2012). After mammary stimulation terminates, females are expected to resume breeding activity rapidly to maximize their reproductive success (van Schaik 2000a). Wolff and Macdonald (2004) found that litter loss induced estrus cycling in 106 (80%) of 133 species of mammals belonging to 33 families and nine orders.

Infanticide can be a male reproductive strategy, that is, sexually selected infanticide (SSI), provided that three conditions are fulfilled (Hrdy 1979). First, an infanticidal male should only kill unrelated and dependent offspring. Second, the infanticide should trigger estrus in the victimized mother and shorten her interlitter interval; and third, the infanticidal male should have a high probability of siring the victimized females' subsequent litter (Trivers 1972; Hrdy 1979). Species vulnerable to SSI are expected to have a longer lactation than gestation period, and exhibit lactational anestrus (van Schaik 2000b). Evidence for SSI is extremely difficult to document in the field, especially for nonsocial species with an elusive lifestyle, and SSI has been documented almost exclusively in social, nonseasonally breeding mammals (Bellemain et al. 2006a). Mathematical modeling suggests that even a small time lag between litter loss and the next conception can make infanticide untenable as a male reproductive strategy (Hrdy and Hausfater 1984). Therefore, SSI has been proposed to be rare among seasonal breeders (Hausfater 1984; Hrdy and Hausfater 1984; Bartos and Madlafousek 1994). However, suggestive evidence for SSI has been found in seasonal breeders, such as red deer (*Cervus elaphus*; Bartos and Madlafousek 1994), Japanese macaques (*Macaca fuscata*; Soltis et al. 2000), white-throated round-eared bats (*Lophostoma silviculum*; Knörnschild et al. 2011), and brown bears (*Ursus arctos*; Swenson et al. 1997; Bellemain et al. 2006a).

The brown bear, our model species, is a large nonsocial carnivore with a polygamous mating system, lactational anestrus, and a breeding season that lasts from late spring to early summer (Steyaert et al. 2012). Infanticide is common in the brown bear (Craighead et al. 1995), and there is evidence for the SSI hypothesis (Swenson et al. 1997; Swenson 2003; Bellemain et al. 2006a), albeit contested (Miller 1990; Miller et al. 2003; McLellan 2005). Bellemain et al. (2006a) found genetic evidence for two requirements of the SSI hypothesis, that is, that males did not kill their own progeny and that presumed perpetrators had a high probability of siring the victimized mother's next litter. Anecdotal observations of mixed-aged litters following short-term family breakups during the mating season (∼3.3 days) suggest that females can rapidly shift to breeding conditions after mammary stimulation ends (requirement II of the SSI hypothesis) (Swenson and Haroldson 2008). However, it remains unclear whether or not these anecdotal observations can be generalized, and how females change reproductive strategies after losing an entire litter.

In this study, we first describe the reproductive fate of female bears that lost an entire litter of dependent offspring during the mating season, based on a long-term demographic dataset of individually monitored bears (1986–2011), and we test whether litter loss shortens interlitter intervals. Secondly, we used Global Positioning System (GPS) relocation data (2005–2011) to evaluate how females changed reproductive strategies after losing an entire litter of dependent offspring during the mating season. We define “litter loss” as the disappearance or death of an entire litter of dependent offspring. We consider “dependent offspring” as cubs-of-the-year (hereafter “cubs”), because older offspring are not necessarily dependent on their mother for their survival (Dahle and Swenson 2003a). We hypothesized that females would shift to mating behavior within a few days (∼3) after litter loss during the mating season. Previous research suggested that female brown bears apply different reproductive strategies depending on their reproductive status, that is, receptive females actively roam-to-mate and that females with cubs (hereafter “females/cubs”) tend to adapt a secretive and sedentary lifestyle, presumably to minimize the risk of infanticide (Dahle and Swenson 2003b; Martin et al. 2013; Steyaert et al. 2013a,b2013b). Therefore, we predicted that (1) females/cubs move less than receptive females during the mating season. As a sedentary lifestyle should be favored by females/cubs to minimize the risk of encountering potentially infanticidal males (Ebensperger 1998; Dahle and Swenson 2003b; Martin et al. 2013), or analogous to predator–prey theory, to avoid spreading scents that could attract infanticidal males (Hughes et al. 2010), we predicted (2) a positive relationship between movement of females/cubs and litter loss. Because we expect that females would enter estrus rapidly after litter loss during the mating season and roam to acquire mates, we predicted (3) that movement patterns of receptive females and females that experience litter loss would become indistinguishable rapidly (a few days) after a litter loss event during the mating season.

## Materials and Methods

### Study area and species

The study was conducted in Dalarna and Gavleborg counties in south-central Sweden (61°N, 15°E). The area is covered with intensively managed boreal forest (>80%, mainly Norway spruce, *Picea abies* and Scots pine, *Pinus sylvestris*), bogs, and lakes. Refer to Martin et al. (2010) for a detailed study area description.

The brown bear is a nonsocial, size-dimorphic large carnivore with a polygamous mating system, and a mating period in late spring and early summer (Spady et al. 2007; Steyaert et al. 2012). Males and receptive females typically expand their home ranges in the mating season and roam to acquire mates (Dahle and Swenson 2003b; Martin et al. 2013). Cubs (typically 2–3) are born during winter denning (Friebe et al. 2013), and lactation can last up to 2.5 years, with a peak during the cubs' first midsummer (Farley and Robbins 1995). Cubs stay with their mother for 1.5–4.5 years, but are not necessarily dependent on their mother after their first year (Dahle and Swenson 2003a). Family breakups occur almost exclusively during the mating season, after which the mothers typically mate, and give birth the subsequent birthing season (Dahle and Swenson 2003a). Interlitter intervals are thus usually approximately 6 months longer than the time that the cubs stay with their mother.

Cub mortality varies among populations and can be as high as 66% (Miller et al. 2003). Most mortality occurs during the mating season and is mostly caused by infanticide by adult males (McLellan 1994; Swenson et al. 1997; Bellemain et al. 2006a; Zedrosser et al. 2009). In our study area, nonparental infanticide explains at least 92% of all the cub loss (16 documented infanticide events, totaling ≥31 killed cubs) during the mating season, and we never recorded females committing infanticide (Bellemain et al. 2006b; Steyaert 2012; Steyaert et al. 2013a). Zedrosser et al. (2009) found that primiparous (generally younger) females more often lose their litter, perhaps because they are less experienced in avoiding infanticidal males and providing offspring defense than multiparous (generally older) females. Females/cubs actively defend their litter (Craighead et al. 1995), use spatiotemporal avoidance of conspecifics during the mating season (Wielgus and Bunnell 1995; Ben-David et al. 2004; Steyaert et al. 2013a,b2013b), and engage in multimale mating (Bellemain et al. 2006b) as counterstrategies to SSI. For a detailed review of the mating system of the brown bear, see Steyaert et al. (2012).

### Bear monitoring

Since 1985, bears were routinely captured and marked with collars bearing very high frequency (VHF) (MOD-500, Telonics Inc., Mesa, AZ) and GPS telemetry devices (GPS Plus collars [Berlin, Germany], Vectronic Aerospace GmbH, from 2003 onwards). Interlitter intervals were documented, and cub presence and survival were monitored from a helicopter or ground three times per year for the entire study period (1986–2012), that is, shortly after den emergence, shortly after the mating season, and prior to denning. In addition, we monitored the presence of cubs with their mothers continuously during the 2008–2011 mating seasons, with surveys from a helicopter, direct observations from the ground, and from tracks and signs of cubs collected at clusters of the mothers' GPS positions (hereafter “cluster sites”, minimum three consecutive GPS positions within a 15-m radius). For details on capture and handling, refer to Arnemo et al. (2011).

### Relocation data and movement rates

The GPS collars were scheduled to take one position every 30 min, thus theoretically fixing 48 positions per day. We removed GPS fixes with a dilution of precision value ≥5, and all two-dimensional (2D) fixes in order to increase spatial accuracy (Lewis et al. 2007). This reduced the average fix success rate from 94% to 73%. To ensure full data coverage during the mating season, we selected data from 1 May to 31 July to examine movement rates. We calculated movement rate (km/h) of an individual bear based on the Euclidean distance between 30-min consecutive GPS locations. We defined three reproductive classes of bears: receptive females (≥5 years and conceived; i.e., emerging from the den with cubs the subsequent year), females/cubs (≥5 years and accompanied with dependent young <1 year old), and females that lost their litter during the mating season (hereafter females/litter loss).

For each prediction, we created a separate dataset, comprised of movement data from receptive females and females/cubs (prediction 1), females/cubs and females/litter loss (before the date of cub loss) (prediction 2), and receptive females and females/litter loss (after the date of cub loss) (prediction 3).

### Statistical analysis

We used a generalized linear mixed effect model with a Poisson error structure to evaluate how litter loss related to the response variable “interlitter interval”. We controlled for “age of the mother” and included “year” and “bear ID” as random model terms. We used generalized additive mixed models (GAMMs) to test the three predictions. We used movement rate as the response variable and included “year” and “bear ID” as random factors. Because brown bears show temporal variation in their behavior (Moe et al. 2007; Martin et al. 2010), we included “time of day” (1–48, 30 min intervals) and “Julian day” (day 1–92, starting from 1 May) for each reproductive status as nonlinear model terms. We used cyclic cubic regression splines to fit “time of day”, because this method connects the beginning and end points of a cycle (here “day”) and thin plate regression splines to fit “Julian day”, in which the beginning and end points of a cycle are not constrained by each other (Zuur et al. 2009).

For predictions 1 and 3, we considered the fixed variables “age”, “reproductive status”, and the interaction term “age × reproductive status” for inclusion in our models. For prediction 2, we considered the fixed variables “primiparity/multiparity”, “reproductive status”, and the interaction term between these two variables for inclusion in our models. For predictions 1 and 3, we could not include “primiparity/multiparity” as a variable, because of singularities. For prediction 2, we did not include “age” as proxy for female experience, because of the close relationship between age and primiparity (Zedrosser et al. 2009). To improve model fit, we included a variance component which allowed heterogeneity among the different reproductive classes in each model. For each prediction, we selected the most parsimonious model from all possible combinations of the three fixed variables (including a null model) based on Akaike's Information Criteria differences (second-order bias corrected, ΔAIC_C_) and weights (AIC_CW_) (Akaike 1973; Anderson 2008). Candidate models with ΔAIC_C_ values <2 are presented in Appendix S1. We used the “mgcv” (Wood 2011) and “lme4” (Bates and Maechler 2010) packages in R 2.14.0 (R Development Core Team 2013) for statistical analysis. We validated the statistical models by plotting model residuals versus the fitted values (Zuur et al. 2009).

## Results

### Long-term demographic data – reproductive fate after litter loss

Between 1986 and 2011, we recorded 268 litters from 92 females. In 42 cases, the fate of the cubs and/or the mother was unknown because the females were killed by either hunters or other bears or were lost due to transmitter failure. From the remaining 226 cases, 149 (66%) litters were weaned successfully, whereas litter loss occurred in 77 (34%) cases. About 82% of all litter loss occurred during the premating and the mating season (April–July). About 15% of the litter losses occurred before April, whereas only about 3% of the litter losses occurred after July (two cases in August and one case in September). Litter loss significantly shortened interlitter intervals (*ß*_survival_ = 0.77, SE = 0.039, *z *=* *19.28, *P* < 0.001), and in 70 (91%) of the 77 cases of litter loss in which the reproductive fate of the mothers was known, the mothers emerged from their den with cubs the subsequent spring.

### GPS relocation data – reproductive fate and behavioral changes after litter loss

We obtained GPS relocation data from 29 females that were monitored during at least one mating season between 2005 and 2011 (*N* = 63 mating seasons). We classified 15 females as “receptive female” during at least one mating season during the study period (*N* = 23). We recorded cub presence throughout the mating season in 29 cases, from 23 different females. We recorded litter loss during the mating season in 11 cases, but could only accurately estimate the date of litter loss in eight cases, from seven different females. In five cases, we obtained precise estimates of date and time of cub death, based on GPS data from the mother and the remains of cubs collected in the field. For the remaining three cases, we are confident to have estimated the correct day of litter loss, because of presence/absence of cub tracks and signs at cluster sites. We recorded movement rates between two valid 30-min relocations in 100,161 cases, averaging 1642 (167–3120) movement rate measures per individual. In 10 of the 11 cases (91%) in which GPS-marked females lost their litter, the females were observed with GPS-marked males during the ongoing mating season (2, 5, 6, 7, 14, 19, and 39 days after losses, in cases of known date of loss). Seven of the 11 (64%) GPS-marked females that lost their litters gave birth during the next birthing period. However, three females were shot during the hunting season prior to the next birthing period, so it is unknown whether they would have reproduced successfully. Therefore, of known outcomes of GPS-marked females, 7/8 (87.5%) females that lost their litter still conceived and gave birth in the next birthing season. One female emerged from her den without cubs-of-the-year following the loss.

#### Prediction 1: females/cubs move less than receptive females during the mating season

**Figure 1:**
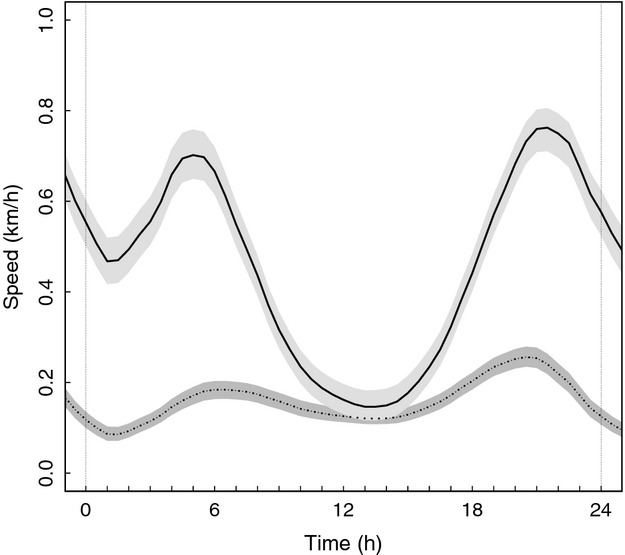
Mean diurnal movement rates (km/h) fitted with a moving average spline of lone female brown bears (―) and females with cubs-of-the-year (....) during the mating season in central Sweden during 2006–2011. The shaded areas represent the 95% pointwise bootstrapped confidence regions around the means. The vertical dashed lines delineate one day, from midnight to midnight.

**Figure 2:**
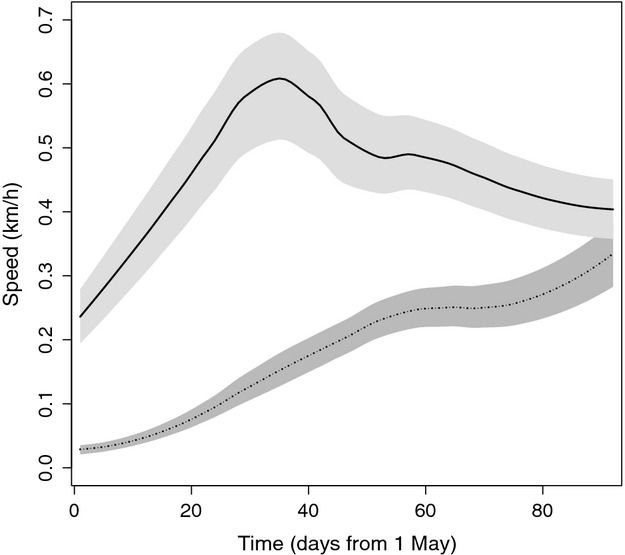
Mean daily movement rates (km/h) fitted with a moving average spline for lone female brown bears (―) and females with cubs-of-the-year (....) during the mating season in central Sweden during 2005–2011. The shaded areas represent the 95% pointwise bootstrapped confidence regions around the means.

The most parsimonious model (AIC_CW_ = 0.46) to test differences in movement rates between receptive females during the mating season (*N* = 23) and females/cubs (*N* = 29) included “reproductive status” and “age” as fixed variables (Table 1). Females/cubs moved less than receptive females (*ß *=* *−0.276, standard error (SE) = 0.016, *t *=* *−17.0, *P *<* *0.001) during the mating season. Age had no significant effect on female movement rates (*ß *=* *−0.0002, SE = 0.002, *t *=* *−0.085, *P *=* *0.932). The regression splines “time of day” (females/cubs, *edf* = 7.855, *F* = 175.61, *P *<* *0.001; receptive females, *edf* = 7.913, *F *=* *542.8, *P *<* *0.001) and “Julian day” (females/cubs, *edf* = 7.467, *F *=* *571.25, *P *<* *0.001; receptive females, *edf* = 8.471, *F *=* *96.98, *P *<* *0.001) affected the movement rates of both reproductive classes (Table 1). Receptive females showed a distinct bimodal diurnal movement pattern, with peaks around 4:00–6:00 and 21:00–22:00 (∼0.7 km/h) and a distinct low (∼0.2 km/h) around midday (Fig. 1). This bimodal pattern was much less distinct for females/cubs, with maximum movement rates around 21:00 at ∼0.25 km/h (Fig. 1). The average movement rate of receptive females during the mating season was 0.46 km/h and peaked around early June (Julian day 30–35) at ∼0.6 km/h. Movement rates of females/cubs averaged 0.16 km/h during the mating season and gradually increased from ∼0.04 km/h in early May to ∼0.23 km/h in late July (Fig. 2). Two candidate models had nearly identical ΔAIC_C_ values <2 (candidate 1, ΔAIC_C_ = 1.0604; candidate 3, ΔAIC_C_ = 1.06, Appendix S1). The results of these two models generally agreed with those of the most parsimonious model (Appendix S1).

**Table 1:** Summary of the most parsimonious GAMMs for the three predictions to test for differences in movement rates among reproductive classes of female brown bears in central Sweden in the mating season during 2006–2011.

Prediction 1: Comparing movement rates between females/cubs and receptive females				
Movement rate ∼ S(time of day) + S(Julian day) + *F*(reproductive state) + *F*(age) + R(year) + R(ID) + V(reproductive state) (AIC_CW_ = 0.46)				
Variable	ß	SE	*t*	*P*
Intercept	0.45	0.029	15.3	<0.001
Reproductive state (females/cubs vs. receptive females)	−0.276	0.016	−17.0	<0.001
Age	−0.0002	0.002	−0.085	0.932
		*edf*	*F*	*P*
Time of day: females/cubs		7.855	175.6	<0.001
Time of day: receptive females		7.913	542.8	<0.001
Julian day: females/cubs		7.467	571.3	<0.001
Julian day: receptive females		8.471	96.98	<0.001
Prediction 2: Comparing movement rates between females/cubs before litter loss and females/cubs				
Movement rate ∼ S(time of day) + S(Julian day) + *F*(reproductive state) + R(year) + R(ID) + V(reproductive state) (AIC_CW_ = 0.49)				
Variable	ß	SE	*t*	*P*
Intercept	0.093	0.007	13.99	<0.001
Reproductive state (females/cubs before litter loss vs. females/cubs)	0.043	0.008	5.125	<0.001
		*edf*	*F*	*P*
Time of day: females/cubs		7.417	57.81	<0.001
Time of day: females/cubs before litter loss		6.095	38.94	<0.001
Julian day: females/cubs		4.757	299.66	<0.001
Julian day: females/cubs before litter loss		7.760	66.54	<0.001
Prediction 3: Comparing movement rates between females/cubs after litter loss and receptive females				
Movement rate ∼ S(time of day) + S(Julian day) + *F*(age) + R(year) + R(ID) + V(reproductive state) (AIC_CW_ = 0.28)				
Variable	ß	SE	*t*	*P*
Intercept	0.355	0.042	8.404	<0.001
Age	0.008	0.004	1.967	0.049
		*edf*	*F*	*P*
Time of day: receptive females		7.913	529.2	<0.001
Time of day: females/cubs after litter loss		7.814	311.1	<0.001
Julian day: receptive females		8.472	97.67	<0.001
Julian day: females/cubs after litter loss		6.564	58.19	<0.001

These reproductive classes are receptive females (≥5 years and conceived; that is, emerging from the den with cubs the subsequent year), females with cubs-of-the-year (>5 year, with cubs-of-the-year, females/cubs), females/cubs before litter loss, and females/cubs after litter loss. We used movement rate (km/h) as the response variable. “Time of day” and “Julian day” were included as regression splines (S), “reproductive status”, “age”, or “primiparity/multiparity” as fixed variables (*F*), “year” and “bear ID” as random components (R), and a variance component (V) that allowed heterogeneity between different levels of reproductive status. Parameter estimates (ß), standard errors (SE), test statistics (*t*), and *P*-values (*P*) are shown for the intercept and the fixed variables. Spline statistics are summarized per “Julian day” and “time of day”, and per reproductive status.

#### Prediction 2: a positive relationship between movement rate and litter loss

**Figure 3:**
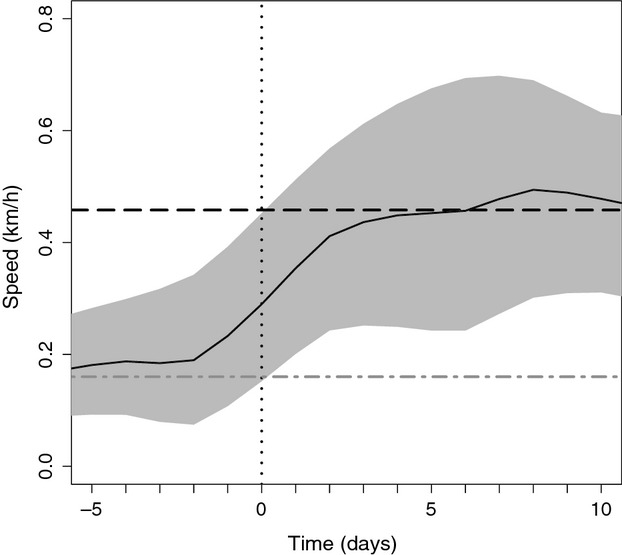
The change in movement rates (km/h) of female brown bears after litter loss (―) in central Sweden, during the mating seasons of 2006–2011. We centered the average daily movements of individual females that had lost their litter at the day of loss (day 0, vertical dashed line). The shaded area represent the 95% pointwise bootstrapped confidence region around the mean daily movement rates of all females that lost litters. The horizontal dashed lines represent seasonal average movement rates of lone females (― ―) and of females that keep their litters throughout the mating season (*- -*).

The most parsimonious model (AIC_CW_ = 0.49) to test for differences in movement rates between females/cubs (*N* = 29) and females/litter loss (*N* = 8) only included “reproductive status” as a fixed variable. Females that had lost their litter moved more before the date of loss than females with litters that survived during the entire mating season (*ß *=* *0.043, SE = 0.008, *t *=* *5.125, *P *<* *0.001) (Table 1) (Fig. 3). Also the splines “time of day” and “Julian day” were significant model terms for females/cubs before the loss (time of day, *edf* = 6.095, *F *=* *38.94, *P *<* *0.001; Julian day, *edf* = 7.76, *F *=* *66.54, *P *<* *0.001) and females/cubs (time of day, *edf* = 7.417, *F *=* *57.81, *P *<* *0.001; Julian day, *edf* = 4.757, *F *=* *299.66, *P *<* *0.001) (Table 1). The average daily movement rates of females/litter loss increased approximately 2 days before the loss (Fig. 3). The candidate model that included “reproductive status” and “primiparity/multiparity” had ΔAIC_C_ and AIC_CW_ values of 1.76 and 0.204, respectively (Appendix S1). Similar to the most parsimonious model, females that had lost their litter moved more before the date of loss than females with litters that survived during the entire mating season (*ß *=* *0.042, SE = 0.009, *t *=* *4.912, *P *=* *0.002), and “primiparity/multiparity” did not affect movement patterns of females before the date of litter loss (*ß *=* *0.004, SE = 0.009, *t *=* *5.125, *P *=* *0.6378) (Appendix S1).

#### Prediction 3: changing strategies after litter loss, from elusive to roam-to-mate behavior

The most parsimonious model (AIC_CW_ = 0.28) to evaluate movement rates of females/litter loss (*N* = 8) and receptive females (*N* = 23) only included the fixed variable “age” (*ß *=* *0.008, SE = 0.004, *t *=* *1.967, *P *=* *0.0492) (Table 1). Movement patterns of receptive females and females that experienced litter loss were not distinguishable, because “reproductive status” was not included in the most parsimonious model. “Time of day” and “Julian day” significantly affected movements of receptive females (time of day, *edf* = 7.913, *F *=* *529.3, *P *<* *0.001; Julian day, *edf* = 8.472, *F *=* *97.67, *P *<* *0.001) and females/litter loss after the event of loss (time of day, *edf* = 7.814, *F *=* *311.1, *P *<* *0.001; Julian day, *edf* = 6.564, *F *=* *58.19, *P *<* *0.001). The average daily movement rates of females that experienced litter loss increased until approximately 4 days after the loss. From 1 day after litter loss, the 95% confidence region of average daily movement rates of females/litter loss included the seasonal average movement rate of receptive females (Fig. 3). Six other candidate models had ΔAIC_C_ values <2, including the null model (Appendix S1). “Reproductive status” was never included as a significant model term in any of the models (−0.129 < *ß *<* *−0.009, 0.03 < SE = 0.074, −1.739 < *t* < −0.309, 0.082 < *P *<* *0.757) (Appendix S1).

We evaluated the “age” effect of the most parsimonious model separately for females after litter loss and receptive females as a post-hoc analysis with GAMMs. After controlling for “time of day” and “Julian day”, “year”, and “bear ID”, we found that age had a strong positive significant effect on movement rates of females that had lost their litter (*ß *=* *0.018, *P *<* *0.001), but not for receptive females (*ß *=* *0.003, *P *=* *0.570). We found no signs of heteroskedasticity in the residuals of any of the models, suggesting good model fit (Zuur et al. 2009).

## Discussion

Our data showed that the loss of an entire litter is common in our study population, and we documented that the majority (91%) of females that experience litter loss during the mating season also enter estrus during the ongoing mating season, mate successfully, and give birth in the subsequent birthing season. This is a conservative estimate, however, because some females gave birth and lost their young in the den (Friebe et al. 2013). We found that litter loss induced a rapid behavioral change, that is, from an elusive lifestyle typical for females/cubs to roam-to-mate behavior typical for receptive females, in no more than 1 day. Already 1 day after litter loss, average daily movement rates of receptive females and females/litter loss were indistinguishable (Fig. 3). Our results show that litter loss shortens interlitter intervals in brown bears, and provide support for the second prediction of the SSI hypothesis.

Patterns in movement data were in accordance with the expected behavior of females/cubs and receptive females, that is, an elusive, sedentary lifestyle to enhance offspring survival and roam-to-mate behavior, respectively. Females/cubs moved on average at about 1/3 of the rate of, and in a less bimodal diurnal pattern than, receptive females during the mating season. An increased movement rate of females/cubs during the mating season increased the risk of litter loss. The average daily movement rates of females/cubs increased gradually during the mating season, whereas daily movement rates of receptive females peaked around early June. This peak in movement rates of receptive females coincided with the “peak” of the mating season (Steyaert 2012), suggesting that receptive females actively roam-to-mate.

The mean interlitter interval in female brown bears varies among populations, from an average 2.4 (Sæther et al. 1998) years in central Sweden to 5.7 years in a high-altitude population in Pakistan (Nawaz et al. 2008). In our study population, at least 91% of the females that experience litter loss during the mating season, also enter estrus, mate, and give birth during the next birthing season, implying that their interlitter interval is shortened to approximately 12 months. Complete litter loss during the mating season can thus shorten interlitter intervals in female brown bears by 50% (in the case of a 2-year interlitter interval) to 80–85% (in the case of a 5-or 6-year interlitter interval). Consequently, infanticide can drastically reduce interlitter intervals in female brown bears and provide a considerable reproductive advantage for infanticidal males. There is a growing body of evidence for SSI in seasonal breeders other than brown bears, for example hanuman langurs (*Presbytis entellus*; Borries 1997), red deer (Bartos and Madlafousek 1994), Japanese macaques (Soltis et al. 2000), white-throated round-eared bats (Knörnschild et al. 2011), ringtail lemurs (*Lemur catta*; Jolly et al. 2000), and patas monkeys (*Erythrocebus patas*; Enstam et al. 2002). van Noordwijk and van Schaik (2000) estimated the vulnerability for SSI of 211 mammals based on their life-history parameters (i.e., breeding and birthing seasonality, lactation and gestation duration, delayed implantation, promiscuity, etc.). They suggested that 142 of these species were vulnerable for SSI and that 41 of these species (mainly carnivores, pinnipeds, and primates) show seasonality in birthing and mating. Thus, SSI is probably more common among mammals with reproductive seasonality than has been observed or reported.

The movement patterns of females/cubs and receptive females supported prediction 1, suggesting that movement rate can be a good correlate for reproductive status (Nathan et al. 2008). Because SSI can occur throughout the mating season, we suggest that the general increase in daily movement rates of females/cubs throughout the mating season may be explained by the internal state of the females and their cubs, that is, increased nutritive requirements by the mothers and their cubs and/or increased mobility of the cubs (Martin et al. 2013). The peak of average daily movement rates of receptive females coincided with the peak of the mating season, supporting the hypothesis that females roam-to-mate as a reproductive strategy (Dahle and Swenson 2003b). Increased and adjusted movement patterns of females in estrus or during the mating season also have been reported in other polygamous species, such as red deer (Stopher et al. 2011), roe deer (*Capreolus capreolus*, San José and Lovari 1998), pronghorn antelope (*Antilocapra americana*, Byers et al. 2005), and polar bears (*Ursus maritimus*, Laidre et al. 2013). Among mammals, females are the choosier sex regarding mates, because they invest more in their offspring than males (Trivers 1972; Andersson 1994). To obtain high-quality mates, receptive females can use active search strategies. Female searching behavior, however, is poorly understood and is rarely documented in the literature (Lovari et al. 2008; Stopher et al. 2011).

Two mechanisms may explain the movement patterns of females/cubs during the mating season. First, reducing activity and movement can be a strategy to reduce predation risk in general (Sih and McCarthy 2002), including the risk of infanticide by conspecifics (Ebensperger 1998; Swenson 2003). Second, the mobility of dependent offspring can be a restricting factor for their mothers' movements. We found that movement rates of females that experienced litter loss were higher before the event of litter loss than those of females that kept their litter throughout the mating season (prediction 2), suggesting that being sedentary is an adaptive strategy to enhance offspring survival. Other research also provides suggestive evidence that females/cubs reduce their movements during the mating season, below that which cubs are capable of, to lower the risk of infanticide (e.g., Dahle and Swenson 2003b; Swenson 2003; Martin et al. 2013), and spatiotemporal infanticide avoidance strategies have been suggested in various brown bear populations (Wielgus and Bunnell 1995; Ben-David et al. 2004; Rode et al. 2006; Steyaert et al. 2013a,b2013b), as well as in primates (Hrdy 1979), rodents (Coulon et al. 1995), cetaceans (Loseto et al. 2006), and felids (Packer and Pusey 1983). Our results are not unambiguous, however, because external factors, such as disturbance by humans or conspecifics also may provoke increased movement rates (i.e., flight) (Moen et al. 2012). Increased movements may expose individuals to greater risks, such as predation and accidents (Lima and Dill 1990), and perhaps lead to abandonment or loss of offspring.

We found that movement rates of receptive females and of females after litter loss were not distinguishable already 1 day after the litter loss (prediction 3). A rapid shift was expected for a seasonal breeder with lactational anestrus and sexually selected infanticide (Weir and Rowlands 1973; Swenson and Haroldson 2008). The second requirement of the sexual selection hypothesis states that, after infanticide, the victimized mothers can be fertilized earlier than if her offspring had survived (Hrdy 1979). Females should return to breeding conditions immediately after litter loss, if it is to be advantageous as a male reproductive strategy in seasonally breeding mammals (Hausfater 1984; Hrdy and Hausfater 1984; van Schaik 2000b).

We found that age significantly affected movement rates of females after litter loss, with older females moving more than younger ones. We suggest that older females are probably more experienced than younger ones (Paitz et al. 2007; Zedrosser et al. 2009) and perhaps roam more actively for mate acquisition to maximize reproductive success after litter loss than younger females. Also, reproductive allocation might be related to age because older females are typically larger than younger ones (Zedrosser et al. 2004), potentially making it energetically more challenging for younger and smaller females to engage in reproduction (Cichoń 2001).

## Conclusions

One requirement of the SSI hypothesis is that killing dependent offspring shortens the interlitter interval of the victims' mother. In the case of brown bears, offspring loss during the mating season can shorten the interlitter interval at least by half and up to 85%. Additional long-term demographic data showed that almost all females that lost their litter during a mating season entered estrus, mated, gave birth, and emerged with cubs from their winter den during the next spring.

We found support for the three predictions for female movement rates in relation to the SSI hypothesis. The movement rates of females/cubs were relatively slow during the mating season and reflected their sedentary lifestyle, which they probably adopted to minimize infanticide risk (Dahle and Swenson 2003b). The movement rates of receptive females were much higher than those of females/cubs, especially during the peak of the mating season, and reflected their roam-to-mate behavior. We found that females that had lost their litters during the mating season moved more before litter loss than females that kept their litters throughout the mating season. The circumstances (disturbances and/or internal factors) under which litter loss occurred were largely unknown and require better documentation, as do the effects of litter loss on female hormonal cycling. Our results demonstrated that litter loss induced a rapid behavioral change in a seasonal breeder with lactational anestrus. This rapid change should be expected in seasonal breeders with lactational anestrus, in which infanticide has evolved as a male reproductive strategy.

Our results complete the three requirements for the sexual selection hypothesis to explain infanticide in the brown bear (Bellemain et al. 2006a). We suggest that infanticide as a male reproductive strategy is more prevalent among seasonally breeding mammals than observed or reported, especially in species with strong sexual selection and life histories similar to those of the brown bear.

## Supporting Information

Appendix S1 Summary of the results of the candidate models with second-order bias corrected ΔAIC values <2 for the three predictions to test for differences in movement rates among reproductive classes of female brown bears in central Sweden in the mating season during 2006–2011.
