# Supplementary material for: Litter loss triggers estrus in a nonsocial seasonal breeder
Source: Ecol Evol. 2014 Jan 3;4(3):300–10. doi: 10.1002/ece3.935 (PMC3925432; doi:10.1002/ece3.935)
Supplement: Appendix S1 — Summary of the results of the candidate models with second-order bias corrected ΔAIC values <2 for the three predictions to test for differences in movement rates among reproductive classes of female brown bears in central Sweden in the mating season during 2006–2011. [file ece30004-0300-sd1.docx]

**Appendix S1**

The following Supporting Information is available for this article online:

Appendix 1: Summary of the results of the candidate models with second order bias corrected ΔAIC values <2 for the three predictions to test for differences in movement rates among reproductive classes of female brown bears in central Sweden in the mating season during 2006-2011. These reproductive classes are: receptive females (>5 years and not nulliparous), females with cubs-of-the-year (>5 year, with cubs-of-the-year, females/cubs), females/cubs before litter loss, and females/cubs after litter loss. We used movement rate (km/h) as the response variable. ‘Time of day’ and ‘Julian day’ were included as regression splines (S), ‘reproductive status’, ‘age’ or ‘primiparity/multiparity’ as fixed variables (F), ‘year’ and ‘bear ID’ as random components (R), and a variance component (V) that allowed heterogeneity between different levels of reproductive status. Parameter estimates (ß), standard errors (se), test statistics (t) and p–values (p) are shown for the intercept and the fixed variables. Spline statistics are summarized per ‘Julian day’ and ‘time of day’, and per reproductive status.

| ***Prediction 1: comparing movement rates between females/cubs and receptive females*** | | | | |
| --- | --- | --- | --- | --- |
| Movement rate ~ S(time of day) + S(Julian day) + F(reproductive state) + F(age) + F(reproductive state * age) + R(year) + R(ID) + V(reproductive state) (ΔAICC = 1.06, AIC_CW_ = 0.27) | | | | |
| **Variable** | **ß** | **se** | **t** | **p** |
| intercept | 0.424 | 0.039 | 11.006 | <0.001 |
| reproductive state (females/cubs loss vs. receptive females) | -0.226 | 0.051 | -4.465 | <0.001 |
| age | 0.002 | 0.003 | 0.592 | 0.0554 |
| reproductive state * age | -0.005 | 0.004 | -1.017 | 0.309 |
|  | **edf** | **F** | **p** |  |
| time of day : receptive females | 7.913 | 545.08 | <0.001 |  |
| time of day : females/cubs | 7.855 | 175.61 | <0.001 |  |
| Julian day : receptive females | 8.471 | 96.96 | <0.001 |  |
| Julian day : females/cubs | 7.468 | 571.02 | <0.001 |  |

| ***Prediction 1: comparing movement rates between females/cubs and receptive females*** | | | | |
| --- | --- | --- | --- | --- |
| Movement rate ~ S(time of day) + S(Julian day) + F(reproductive state) + F(reproductive stage * age) + R(year) + R(ID) + V(reproductive state) (ΔAIC_C_ = 1.06, AIC_CW_ = 0.27) | | | | |
| **Variable** | **ß** | **se** | **t** | **p** |
| intercept | 0.425 | 0.039 | 11.006 | <0.001 |
| reproductive state (females/cubs vs. receptive females) | -0.226 | 0.051 | -4.465 | <0.001 |
| receptive females * age | 0.002 | 0.003 | 0.592 | 0.554 |
| females/cubs * age | -0.003 | 0.003 | -0.763 | 0.445 |
|  | **edf** | **F** | **p** |  |
| time of day : receptive females | 7.913 | 545.1 | <0.001 |  |
| time of day : females/cubs | 7.855 | 175.6 | <0.001 |  |
| Julian day : receptive females | 8.471 | 95.5 | <0.001 |  |
| Julian day : females/cubs | 7.468 | 571 | <0.001 |  |

| ***Prediction 2: comparing movement rates between females/cubs before litter loss and females/cubs*** | | | | |
| --- | --- | --- | --- | --- |
| Movement rate ~ S(time of day) + S(Julian day) + F(reproductive state) + F(reproductive state) + R(year) + R(ID) + V(reproductive state) (ΔAIC_C_ = 1.76, AIC_CW_ = 0.204) | | | | |
| **Variable** | **ß** | **se** | **t** | **p** |
| intercept | 0.11 | 0.016 | 6.988 | <0.001 |
| reproductive state (females/cubs before litter loss vs. females/cubs) | 0.044 | 0.009 | 5.115 | <0.001 |
| age | -0.002 | 0.001 | -1.241 | 0.215 |
|  | **edf** | **F** | **p** |  |
| time of day : females/cubs | 7.418 | 57.84 | <0.001 |  |
| time of day : females/cubs before litter loss | 6.096 | 38.94 | <0.001 |  |
| Julian day : females/cubs | 4.766 | 298.35 | <0.001 |  |
| Julian day : females/cubs before litter loss | 7.758 | 66.63 | <0.001 |  |

| ***Prediction 3: comparing movement rates between females/cubs after litter loss and receptive females*** | | | | |
| --- | --- | --- | --- | --- |
| Movement rate ~ S(time of day) + S(Julian day) + F(reproductive state) + F(age) + F(reproductive state * age) + R(year) + R(ID) + V(reproductive state) (ΔAIC_C_ = 1.93, AIC_CW_ = 0.107) | | | | |
| **Variable** | **ß** | **se** | **t** | **p** |
| intercept | 0.414 | 0.049 | 8.51 | <0.001 |
| reproductive state (females/cubs after litter loss vs. receptive females) | -0.129 | 0.074 | -1.739 | 0.0821 |
| age | 0.003 | 0.004 | 0.738 | 0.461 |
| reproductive state * age | 0.012 | 0.007 | 1.65 | 0.099 |
|  | **edf** | **F** | **p** |  |
| time of day : receptive females | 7.913 | 529.2 | <0.001 |  |
| time of day : females/cubs after litter loss | 7.814 | 311.15 | <0.001 |  |
| Julian day : receptive females | 8.472 | 97.64 | <0.001 |  |
| Julian day : females/cubs after litter loss | 6.575 | 57.75 | <0.001 |  |

| ***Prediction 3: comparing movement rates between females/cubs after litter loss and receptive females*** | | | | |
| --- | --- | --- | --- | --- |
| Movement rate ~ S(time of day) + S(Julian day) + F(reproductive state) + F(age) + R(year) + R(ID) + V(reproductive state) (ΔAIC_C_ = 1.92, AIC_CW_ = 0.107) | | | | |
| **Variable** | **ß** | **se** | **t** | **p** |
| intercept | 0.361 | 0.045 | 8.089 | <0.001 |
| reproductive state (females/cubs after litter loss vs. receptive females) | -0.009 | 0.03 | -0.309 | 0.757 |
| age | 0.007 | 0.004 | 1.869 | 0.062 |
|  | **edf** | **F** | **p** |  |
| time of day : receptive females | 7.913 | 554.62 | <0.001 |  |
| time of day : females/cubs after litter loss | 7.814 | 326.6 | <0.001 |  |
| Julian day : receptive females | 8.472 | 97.67 | <0.001 |  |
| Julian day : females/cubs after litter loss | 6.575 | 57.8 | <0.001 |  |

| ***Prediction 3: comparing movement rates between females/cubs after litter loss and receptive females*** | | | | |
| --- | --- | --- | --- | --- |
| Movement rate ~ S(time of day) + S(Julian day) + F(reproductive state) + F(reproductive stage * age) + R(year) + R(ID) + V(reproductive state) (ΔAIC_C_ = 1.93, AIC_CW_ = 0.107) | | | | |
| **Variable** | **ß** | **se** | **t** | **p** |
| intercept | 0.414 | 0.049 | 8.51 | <0.001 |
| reproductive state (females/cubs after litter loss vs. receptive females) | -0.129 | 0.074 | -1.739 | 0.082 |
| receptive females * age | 0.003 | 0.004 | 0.738 | 0.461 |
| females/cubs after litter loss * age | 0.015 | 0.006 | 2.394 | 0.017 |
|  | **edf** | **F** | **p** |  |
| time of day : receptive females | 7.913 | 554.62 | <0.001 |  |
| time of day : females/cubs after litter loss | 7.814 | 311.15 | <0.001 |  |
| Julian day : receptive females | 8.472 | 97.64 | <0.001 |  |
| Julian day : females/cubs after litter loss | 6.575 | 57.75 | <0.001 |  |

| ***Prediction 3: comparing movement rates between females/cubs after litter loss and receptive females*** | | | | |
| --- | --- | --- | --- | --- |
| Movement rate ~ S(time of day) + S(Julian day) + F(age) + F(reproductive state * age) + R(year) + R(ID) + V(reproductive state) (ΔAIC_C_ = 1.96, AIC_CW_ = 0.105) | | | | |
| **Variable** | **ß** | **se** | **t** | **p** |
| intercept | 0.353 | 0.042 | 8.37 | <0.001 |
| age | 0.007 | 0.004 | 1.952 | 0.051 |
| reproductive state * age | 0.001 | 0.003 | 0.2 | 0.842 |
|  | **edf** | **F** | **p** |  |
| time of day : receptive females | 7.913 | 554.66 | <0.001 |  |
| time of day : females/cubs after litter loss | 7.814 | 326.64 | <0.001 |  |
| Julian day : receptive females | 8.472 | 97.66 | <0.001 |  |
| Julian day : females/cubs after litter loss | 6.563 | 57.83 | <0.001 |  |

| ***Prediction 3: comparing movement rates between females/cubs after litter loss and receptive females*** | | | | |
| --- | --- | --- | --- | --- |
| Movement rate ~ S(time of day) + S(Julian day) + F(reproductive state * age) + R(year) + R(ID) + V(reproductive state) (ΔAIC_C_ = 1.96, AIC_CW_ = 0.105) | | | | |
| **Variable** | **ß** | **se** | **t** | **p** |
| intercept | 0.534 | 0.042 | 8.37 | <0.001 |
| receptive females * age | 0.008 | 0.004 | 1.952 | 0.051 |
| females/cubs after litter loss * age | 0.008 | 0.005 | 1.784 | 0.075 |
|  | **edf** | **F** | **p** |  |
| time of day : receptive females | 7.913 | 554.66 | <0.001 |  |
| time of day : females/cubs after litter loss | 7.814 | 326.64 | <0.001 |  |
| Julian day : receptive females | 8.472 | 97.66 | <0.001 |  |
| Julian day : females/cubs after litter loss | 6.563 | 57.83 | <0.001 |  |

| ***Prediction 3: comparing movement rates between females/cubs after litter loss and receptive females*** | | | | |
| --- | --- | --- | --- | --- |
| Movement rate ~ S(time of day) + S(Julian day) + F(reproductive state * age) + R(year) + R(ID) + V(reproductive state) (ΔAIC_C_ = 1.60, AIC_CW_ = 0.126) | | | | |
| **Variable** | **ß** | **se** | **t** | **p** |
| intercept | 0.431 | 0.017 | 24.93 | <0.001 |
|  | **edf** | **F** | **p** |  |
| time of day : receptive females | 7.913 | 554.58 | <0.001 |  |
| time of day : females/cubs after litter loss | 7.814 | 311.11 | <0.001 |  |
| Julian day : receptive females | 8.472 | 97.67 | <0.001 |  |
| Julian day : females/cubs after litter loss | 6.552 | 57.84 | <0.001 |  |
